# Mutational screening of *VSX1*, *SPARC*, *SOD1*, *LOX*, and *TIMP3* in keratoconus

**Published:** 2011-09-24

**Authors:** Patrizia De Bonis, Antonio Laborante, Costantina Pizzicoli, Raffaella Stallone, Raffaela Barbano, Costanza Longo, Emilio Mazzilli, Leopoldo Zelante, Luigi Bisceglia

**Affiliations:** 1 Unità di Genetica Medica, IRCCS Casa Sollievo della Sofferenza, San Giovanni Rotondo, Italy; 2 Unità di Oculistica, IRCCS Casa Sollievo della Sofferenza, San Giovanni Rotondo, Italy; 3 Unità di Oculistica, Università di Foggia, Foggia, Italy; 4 Laboratorio di Oncologia, IRCCS Casa Sollievo della Sofferenza, San Giovanni Rotondo, Italy

## Abstract

**Purpose:**

To evaluate the involvement of Visual System Homeobox 1 (*VSX1*), Secreted Protein Acidic and Rich in Cysteine (*SPARC*), Superoxide Dismutase 1 (*SOD1*), Lysyl Oxidase (*LOX*), and Tissue Inhibitor of Metalloproteinase 3 (*TIMP3*) in sporadic and familial keratoconus.

**Methods:**

Mutational analysis of the five genes was performed by sequencing and fragment analysis in a large cohort of 302 Italian patients, with a diagnosis of keratoconus based on clinical examination and corneal topography. The variants identified in *VSX1* and *SPARC* were also assessed in the available relatives of the probands.

**Results:**

A novel mutation p.G239R and previously reported mutations were found in *VSX1*. Novel and already reported variants were identified in *SPARC* and *SOD1*, whose pathogenic significance has not been established. No pathogenic variants have been identified in *LOX* and *TIMP3*.

**Conclusions:**

Molecular analysis of the five genes in a cohort of 225 sporadic and 77 familial keratoconus cases confirms the possible pathogenic role of *VSX1* though in a small number of patients; a possible involvement of *LOX* and *TIMP3* could be excluded; and the role played by *SOD1* and *SPARC* in determining the disease as not been definitively clarified. Further studies are required to identify other important genetic factors involved in the pathogenesis and progression of the disease that in the authors’ opinion, and according with several authors, should be considered as a complex disease.

## Introduction

Keratoconus (KC; OMIM 148300) is a frequent corneal disease characterized by a progressive conical protrusion of the cornea and no inflammatory central stroma thinning. It is a major indication for corneal transplantation in the Western world [1,2], with a prevalence of approximately 1:2,000 individuals [3,4]. The disease arises in the teenage years with progressive myopia and astigmatism [4] until the fourth decade of life, when it usually stabilizes.

Keratoconus commonly develops as an isolated defect. It can also be one of the findings in syndromic conditions, such as Ehlers-Danlos, Marfan, Apert, Noonan, Down syndromes and, less frequently, in other corneal dystrophies [5]. In the majority of cases, the disease appears sporadic, nevertheless familial transmission have been demonstrated in 6% to 26% of cases [4,6-9], most commonly with an autosomal dominant mode of inheritance with reduced penetrance. Other modes of inheritance have been described, including autosomal recessive mode with variable expressivity, multifactorial inheritance and a major gene model [6,10].

The disease can be diagnosed by well recognized clinical signs, including stromal thinning, Vogt’s striae, Fleischer’s ring, and scissoring of the retinoscopic reflex with a fully-dilated pupil. The most sensitive and accurate diagnostic method is the computer-assisted videokeratography that allows for the detection of keratoconus at an early stage, before clinical signs are evident [11,12].

In a review, Sherwin and Brookes [13] discussed the morphological changes observed in the different structures of keratoconic corneas (epithelium, basement membrane, nerve fibers, Bowman’s layer, stroma, Descemet membrane, endothelium) and concluded that “The diversity of pathology described in keratoconus is likely to represent temporal differences in the progression of the disease, positional differences relative to the apical centre of maximum damage and possibly reflect a variety of pathophysiological diseases that make up the clinical phenotype we identify as keratoconus.” This view seem to be supported by a great number of observations based on biochemical, genetic and expression studies suggesting a possible involvement of several genes and proteins which regulate cellular and extra cellular processes such as proteolysis enzyme activity, wound healing, keratocytes proliferation, differentiation and apoptosis, increased oxidative damage (reviewed by Romero-Jiménez [14]).

Despite the extensive research completed over the last few decades regarding the etiology and the pathogenesis of keratoconus, the cause(s) remain poorly understood. Up to now, Visual System Homeobox 1 (*VSX1*) is the unique gene indicated as a candidate gene in developing the disease. Its nucleotide variants have been analyzed in several populations [15-26], but their pathogenetic role has still not been definitively established.

Other genes, for different reasons, could be considered as candidate genes for keratoconus.

*LOX* (OMIM: 153455) encodes for the protein-lysine 6-oxidase, an extra cellular copper enzyme that initiates the crosslinking of collagens and elastin by catalyzing oxidative deamination of the epsilon-amino group in certain lysine and hydroxylysine residues of collagens and lysine residues of elastin. *SPARC* (OMIM: 182120), encodes for the Secreted Protein Acidic and Rich in Cysteine/osteonectin/BM40, a matrix-associated protein that elicits change in cell shape, inhibits cell-cycle progression, and influences the synthesis of the extracellular matrix (ECM) [27]. *LOX* and *SPARC* are localized on chromosome 5q23.2 and 5q31.3-q32, respectively, in regions that show a suggestive linkage in familial keratoconus [9,28] and thus are both indicated as possible candidate genes for keratoconus.

The Tissue Inhibitors of Metalloproteinases (TIMPs) are the natural inhibitors of matrix metalloproteinases (MMPs), a group of zinc-dependent endopeptidases that exist in both secreted and membrane-bound forms. The balance between MMPs and TIMPs regulates remodeling of the ECM and thus plays a key role in a wide range of physiologic processes that include embryonic development, connective tissue remodeling, wound healing, glandular morphogenesis, and angiogenesis. Because matrix degrading enzymes could potentially influence keratoconus progression, Matthews et al. [29] studied the effects of TIMPs on stromal cell viability and observed that the overexpression of TIMP3 (OMIM: 188826) induced apoptosis in corneal stromal cell cultures. In addition, a more recent study based on cDNA microarrays, showed that TIMP3 was differentially expressed in keratoconic corneas [30].

The Superoxide Dismutase Isoenzymes (SODs) are differently distributed within human healthy cornea and cornea of patients with KC [31]. *SOD1* (OMIM: 147450) is located on chromosome 21 while Trisomy 21 is notably at high risk for keratoconus; thus, a role in the increased oxidative damage found in keratoconic corneas could not be discarded. Udar et al. [32] screened this gene in 15 unrelated patients and identified a 7-base genomic deletion within intron 2 in two of them. Moreover, mRNA analysis showed the presence of two additional transcript splice variants coding for proteins lacking the active site of the SOD1 enzyme.

In this study, we report the mutational analysis results for *VSX1*, *LOX*, *SPARC*, *TIMP3*, and *SOD1*, performed in a large cohort of Italian subjects affected by sporadic and familial keratoconus.

## Methods

### Patients

A total of 302 unrelated probands affected by keratoconus from southern Italy were recruited at the Medical Genetics Unit of IRCCS Hospital Casa Sollievo della Sofferenza (San Giovanni Rotondo, Italy).

Two hundred twenty-five (74.5%) were sporadic cases, whereas the remaining 77 (25.5%) belonged to families in which there were: two affected individuals in 53 pedigrees, three individuals in 15 pedigrees, four individuals in five pedigrees, five individuals in three pedigrees, and six in one pedigree. Eighty-four percent of the probands had bilateral and 16% had unilateral keratoconus. The age of onset/first diagnosis of the disease ranged between 8 and 63 years, with a mean (±SD) age of 25±9.5 and 28±9 years observed for bilateral and unilateral keratoconus patients, respectively.

The diagnosis was based on slit-lamp biomicroscopy and videokeratographic evaluation by using the OPD-Scan ARK-10000 (NIDEK, Tokyo, Japan) and ALLEGRO Oculyzer (WAVELIGHT AG, Erlangen, Germany). In addition, individuals who had a history of the penetrating keratoplasty or other corneal surgical treatments for keratoconus were included in the patient cohort. When determining the familial segregation of the sequence variants, all available family members underwent a clinical examination and corneal topography to examine videokeratographic anomalies typical of clinical or subclinical keratoconus, according to the criteria of Rabinowitz [33] and Levy et al. [8]. In addition, the thinnest point on corneal pachymetry was recorded. Appropriate informed consent was obtained from each subject, and the study was performed according to the tenets of the Declaration of Helsinki. Two-hundred subjects from the general population were enrolled as controls. Eighty patients were previously studied and described [16].

### DNA sequencing analysis

DNA was extracted from peripheral blood leukocytes by standard phenol-chloroform methodology. Each DNA fragment was amplified by polymerase chain reaction (PCR) in a final volume of 25 μl by using 100 ng of genomic DNA. Primers used to amplify *VSX1* exons were those previously published [16]. *LOX, SPARC*, *TIMP3*, and *SOD1* were amplified using custom primers, flanking each exon by at least 50 nucleotides, designed using Primer 3 ([34]; Table 1). All the amplified products were sequenced in both forward and reverse directions, according to dye terminator chemistry and analyzed on an ABI 3130xl Genetic Analyzer (Applied Biosystems, Foster City, CA). cDNA sequencing was used for numbering (GeneBank accession number *VSX1*: NM_014588; *SPARC*: NM_003118; *SOD1*: NM_000454; *TIMP3*: NM_000362; and *LOX*: NM_001178102), where +1 corresponds to the A of the ATG translation initiation codon in the reference sequence.

**Table 1:** Primers used for *LOX, SPARC*, *TIMP3*, and *SOD1* amplification.

**Primer name**	**Sequence**	**TM (°C)**	**Product size**
VSX1_1F	5’-CAGCTGATTGGAGCCCTTC-3’	58	599
VSX1_1R	5’-CTCAGAGCCTAGGGGACAGG-3’		
VSX1_2F	5’-GCACTAAAAATGCTGGCTCA-3’	59	393
VSX1_2R	5’-GCCTCCTAGGAACTGCAGAA-3’		
VSX1_3F	5’-CATTCAGAGGTGGGGTGTT-3’	59	419
VSX1_3R	5’-TCTTGTGGTGCCTTCAGCTA-3’		
VSX1_4F	5’-CGTTGCTTTGCTTTGGAAAT-3’	59	394
VSX1_4R	5’-CGTTGCTTTGCTTTGGAAAT-3’		
VSX1_5F	5’-CCCCAGAGATAGGCACTGAC-3’	59	495
VSX1_5R	5’-TGGACAATTTTTGTCTTTTGG-3’		
SPARC_2F	5’-TTGCACATATCAGGAATTCAG-3’	58	250
SPARC_2R	5’-AGTCCCTGTTCCCTTTCAG-3’		
SPARC_3F	5’-AAGCTCCCCTAGCCTGTATC-3’	60	250
SPARC_3R	5’-TAGCATTGAGACCCACAGC-3’		
SPARC_4F	5’-ACCTGGAACCCTTCAGCTA-3’	58	248
SPARC_4R	5’-CTCATGTAGGCTGTCCTCGT-3’		
SPARC_5F	5’-CCCTGAGATCTGTCCAGGTA-3’	58	294
SPARC_5R	5’-ATAGAACCACCAAGCCAACA-3’		
SPARC_6F	5’-CAGTGTCCCCATCTCTGAA-3’	58	290
SPARC_6R	5’-GGTGGCAGAGACAGCATC-3’		
SPARC_7F	5’-ACCAATGCAGGTGGTATGT-3’	58	348
SPARC_7R	5’-GCTCAGGGGTAAATGCAC-3’		
SPARC_8F	5’-CATGGACCTCTTGTCACACA-3’	58	285
SPARC_8R	5’-AGGGCTTGGAGCAGTATAGG-3’		
SPARC_9F	5’-AAATATCCTTTCCTCCATGCT-3’	58	377
SPARC_9R	5’-AGGCAGAGAGGACAGACAAC-3’		
SPARC_10F	5’-TTGCATGGCCACCTAGAC-3’	58	246
SPARC_10R	5’-CTCCAGGCAGAACAACAAAC-3’		
SOD1_2F	5’-CAGAAACTCTCTCCAACTTTGC-3’	59	218
SOD1_2R	5’-GAGGGGTTTTAACGTTTAGGG-3’		
LOX_1AF	5’-GAGACTGAGATACCCGTGCT-3’	62	474
LOX_1AR	5’-AGCGGTGACTCCAGATGA-3’		
LOX_1BF	5’-TCACAGTACCAGCCTCAGC-3’	62	500
LOX_1BR	5’-ATAGCTGGGGACCAGGTG-3’		
LOX_2F	5’-TTTTCACATTGCTTTGCAGT-3’	56	398
LOX_2R	5’-GCTCTTGTCCCACTTCCTAA-3’		
LOX_3F	5’-TAGTTGGGAAAGGAGGATTG-3’	58	355
LOX_3R	5’-GCAATTTTCTCCCTTCAGGT-3’		
LOX_4F	5’-GACTTATGTCCTGGGGAAAA-3’	56	441
LOX_4R	5’-GATAAAAATGTGTGTGCTCTTCA-3’		
LOX_5F	5’-GGAGGTGCTATAAGGCTGAG-3’	58	370
LOX_5R	5’-TTGCTTCCAATACCATGATT-3’		
LOX_6F	5’-TTCAGGGGAAAATATGCAGT-3’	56	394
LOX_6R	5’-TGCTTACAAGAAAGCTGCTG-3’		
LOX_7AF	5’-CTTAGGTGGAGGGAAACTGT-3’	58	485
LOX_7AR	5’-AAGTCATTTTGGCTCATTCA-3’		
LOX_7BF	5’-GCACATAACTGGATTTTGAACG-3’	56	343
LOX_7BR	5’-TCAGCACCAGATGTGTCCAT-3’		
TIMP3_PrF	5’-AGGGGTAGCAGTTAGCATTC-3’	60	516
TIMP3_PrR	5’-AGGAGGAGGAGAAGCCGT-3’		
TIMP3_1F	5’-ACGGCAACTTTGGAGAGG-3’	60	273
TIMP3_1R	5’-GGGGCAGAGGAAAGGAGT-3’		
TIMP3_2F	5’-CAATTCCAGGCTCCACAGAG-3’	60	300
TIMP3_2R	5’-CTGGCTGGTGCTTAGACACA-3’		
TIMP3_3F	5’-ACATACCCAGCAGTGGGATT-3’	60	296
TIMP3_3R	5’-ACTGGACATTTGGTGAGTCAA-3’		
TIMP3_4F	5’-GGCTAGGCTCTGGACAAAAC-3’	56	248
TIMP3_4R	5’-GCATTGGGAGCTGATGTTTC-3’		
TIMP3_5F	5’-GTCTGAATCCAGGCTCGGTA-3’	56	387
TIMP3_5R	5’-TTTGCAAGAAAACAGGCACT-3’		

### Fragment analysis

To test for the presence of the intronic 7-base deletion (c.169+50delTAAACAG**)** in *SOD1*, genomic DNA was PCR amplified using the forward primer Int2F:5′-CAG AAA CTC TCT CCA ACT TTG C-3′, fluorescently labeled with FAM (Primm, San Raffaele Biomedical Science Park, Milano, Italy) and the reverse primer Int2R:5′-GAG GGG TTT TAA CGT TTA GGG-3′. The 218bp amplified fragment was loaded on an ABI 3100 Genetic Analyzer and analyzed with GeneMapper v4.0 software (Applied Biosystems). Confirmatory sequencing of the deleted fragment was performed after re-amplification of the DNA sample with an identical forward primer IntF2 (fluorescently unlabeled) and Int2R.

### Methylation analysis

One-microgram of genomic DNA purified by standard phenol-chloroform methodology from three normal control and three keratoconic corneal tissues, was subjected to bisulfite treatment and DNA purification using the Epitect Bisulfite kit (Qiagen GmbH, Hilden, Germany) according to the manufacturer’s instructions. Bisulfite-modified DNA from the same treatment was used as a template for fluorescence-based real-time quantitative Methylation-Specific PCR (QMSP). Real-time PCR using a relative quantification method with standard curve to determine methylation levels was performed using the following *TIMP3* primer/probe set: forward 5′-GCG TCG GAG GTT AAG GTT GTT-3′, reverse 5′-CTC TCC AAA ATT ACC GTA CGC G-3′; probe 5′-FAM-AAC TCG CTC GCC CGC CGA A-T AMR A-3′. As reference–gene, a primer/probe set specifically for the unmethylated promoter region of the beta-actin gene (*ACTB*) was used: forward 5′-TGG TGA TGG AGG AGG TTT AGT AAG T-3′; reverse 5′-AAC CAA TAA AAC CTA CTC CTC CCT TAA-3′; probe 5′-FAM-CCA CCA CCC AAC ACA CAA TAA CAA ACA CA-TAMRA-3′. Calibration curves for target and reference genes were constructed using serial dilutions (90–0.009 ng) of a commercially available fully methylated DNA (CpGenome Universal Methylated DNA; Serologicals Corp., Norcross, GA). Amplification reactions were performed in triplicate in a volume of 20 µl that contained 50 ng bisulfite-modified DNA, 600 nM forward and reverse primers, 200 nM probe, 0.6 U of Platinum Taq polymerase (Invitrogen, Inc., Rockville, MD), 200 µM each of dATP, dCTP, dGTP, dTTP, and 2 µl of PCR buffer [35]. PCR conditions were as follows: one step at 95 °C for 3 min, 50 cycles at 95 °C for 15 s, and 60 °C to 62 °C for 1 min. PCR reactions were performed in 96 well plates on an ABI PRISM 7700 Sequence detection system (Applied Biosystems) and were analyzed by SDS 2.1.1 software (Applied Biosystems). Each plate included calibration curves for the *ACTB* and target genes, DNA samples, positive controls (CpGenomeTM Universal Methylated DNA; Serologicals Corp.) and negative controls (Universal Unmethylated DNA; Serologicals Corp.), and multiple water blanks. The relative level of methylated DNA was determined as a ratio of TIMP3 to *ACTB* and then multiplied by 1,000 for easier tabulation. QMSP analysis was repeated for each sample on three separate plates and median values were considered for statistical analyses.

### DHPLC analysis

Denaturing HPLC analysis, using a nucleic acid fragment analysis system (Wave, 3500 HT; Transgenomic, Crewe, UK), was executed to exclude the presence of the *VSX1* and *SPARC* nucleotide variations in control subjects as previously described [16].

## Results

### VSX1

**Figure 1:**
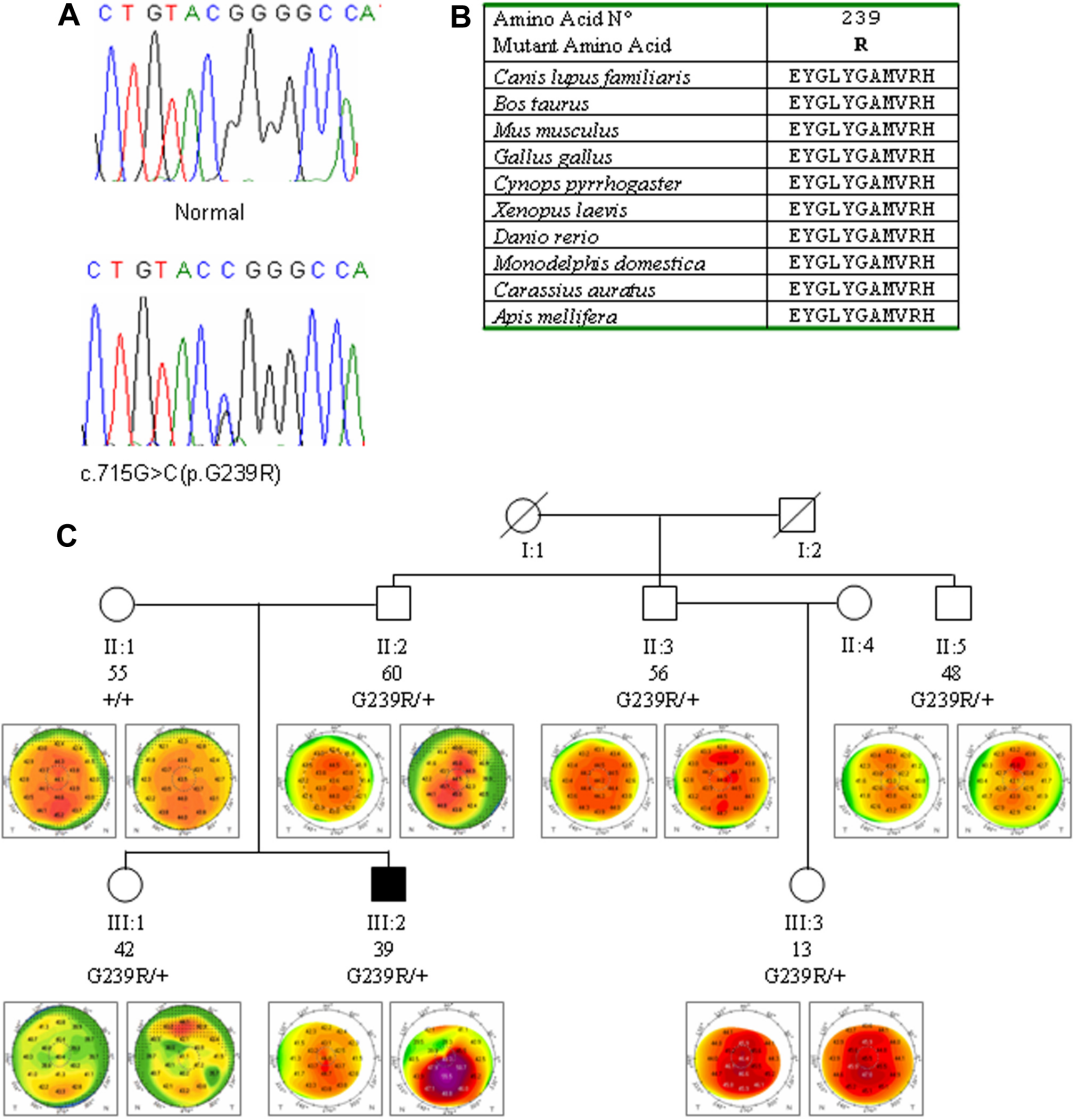
Analysis of the c.715G>C (p.G239R) sequence variant in *VSX1* exon 4. **A**: DNA sequence electropherogram of the c.715G>C (p.G239R) sequence variant in *VSX1* exon 4 (NM_014588). **B**: multiple sequence alignment of the amino acid sequences of VSX1 in different species. Alignments were performed using the program Clustal (provided in the public domain by European Bioinformatics Institute, European Molecular Biology Laboratory, Heidelberg, Germany). **C**: segregation of p.G239R in family K264. Each individual was reported by age (in years), genotype and videokeratographs. Filled symbols refer to keratoconus individual, whereas open symbols are individuals without clinical keratoconus.

The whole coding region and the exon–intron junctions of *VSX1* were analyzed for mutations in 222 of the 302 subjects. A novel heterozygous nucleotide change c.715G>C leading to the substitution of the amino acid glycine with an arginine at the codon 239 (p.Gly239Arg), was identified in the subject K264 (Figure 1A). PolyPhen [36] and SIFT [37] tool analysis predicted that the replaced amino acid would be potentially deleterious (Table 2). The substitution p.Gly239Arg localized in the CVC domain of the VSX1 protein, represents an alteration of an amino acid that has been well conserved across the species (Figure 1B). DHPLC analysis excluded the presence of this variant in 200 normal control individuals. Molecular screening of the p.Gly239Arg substitution was performed in all the family members available. The heterozygous nucleotide change c.715G>C identified in the patient was seen in the father and in other relatives, all showing some quantitative corneal indices altered (Table 3, Figure 1C).

**Table 2:** In silico analysis of novel *VSX1* and *SPARC* variants.

**Gene**	**Protein alteration**	**Polyphen analysis (PSIC score)**	**SIFT analysis (Score)**	**Prediction**
*VSX1*	p.G239R	2.172	<0.05	Probably damaging/deleterious
*SPARC*	p.E63K	0.63	0.13	Benign/tolerated
*SPARC*	p.M92I	2.023	0.45	Probably damaging/tolerated
*SPARC*	p.D219E	0.827	0.16	Benign/tolerated

The prediction of functional impact of missense changes identified was performed using two homology based programs PolyPhen (Polymorphism Phenotyping) [37] and SIFT (Sorting Intolerant From Tolerant) [38] analysis tool. PSIC: (Position Specific Independent Counts).

**Table 3:** Quantitative and qualitative videokeratographic parameters evaluated on keratoconus patients and their relatives.

**Subject ID**	**Age (years)**	**Eye**	**K**	**AST**	**Abs (I-S)**	**Srax**	**KISA**	**Pachymetry**	Corneal shape *
**Family K264**									
II:1	55	OD	44.10	0.9	0.36	1	4.8	525	H
		OS	43.50	0.7	0.42	32	136.4	522	J
II:2	60	OD	43.60	0.8	0.5	20	116.3	539	Jinv
		OS	44.50	2.8	0.48	23	458.5	550	J
II:3	56	OD	44.20	0.4	0.6	1	3.5	503	B
		OS	44.30	0.4	0.26	1	1.5	505	B
II:5	48	OD	43.00	1.6	0.3	15	103.2	524	I
		OS	43.10	2	0.7	15	301.7	517	I
III:1	42	OD	40.2	1.3	-	-	-	517	-
		OS	40.1	1.3	-	-	-	495	-
III:2	39	OD	44.00	1.1	1.32	30	638.9	508	J
		OS	48.30	4.1	9	60	33645	445	D
III:3	13	OD	46.40	1.1	0.32	20	108.9	566	J
		OS	45.50	0.7	0.98	1	10.4	556	F
**Family K361**									
I:1	45	OD	43.72	1.62	2.89	60	4093	558	D
		OS	43.94	1.38	1.67	55	1856	548	D
I:2	43	OD	43.50	2.07	2.95	75	6641	491	D
		OS	43.10	1.31	3.02	60	3410	489	D
II:1	16	OD	42.82	0.77	0.89	27	264	505	J
		OS	43.72	0.89	0.69	23	206	504	J
II:2	14	OD	45.85	0.75	0.48	18	99	543	G
		OS	45.43	1.95	0.32	2.5	24	535	J
II:3	11	OD	43.10	0.76	0.28	7	21	672	F
		OS	43.05	0.71	0.04	5	2	674	F
II:4	9	OD	45.36	0.98	0.84	11	137	529	J
		OS	45.46	1.28	0.16	13	40	527	J

OD=right eye; OS=left eye; K=central keratometry; AST=regular corneal astigmatism; Abs (I-S)=absolute value of inferior-superior dioptric asymmetry; Srax=skewed radial axis index; KISA=K x Abs(I-S) x AST x Srax/3; Pachymetry refers to the thinnest point on the cornea; *corneal shapes classification as reported by Levi et al. [9].

In addition, previously reported amino acid substitutions p.L17P, p.D144E, p.P247R, p.G160D were identified in 9 patients (Table 4). The D144E variant was also found in 1 out of 200 controls.

**Table 4:** Mutational analysis of *VSX1*, *SPARC*, and *SOD1* in 302 subjects affected by keratoconus.

**Gene**	**Patient ID**	**Nucleotide change**	**Protein alteration**	**Controls**
*VSX1*	K33*, K74*, K75*, K124, K295#	c.50T>C	p.L17P	0/200
	K5*, K66*, K96, K161, K305	c.432C>G	p.D144E	1/200
	K35*, K74*, K179	c.479G>A	p.G160D	0/200
	K264	c.715G>C	p.G239R§	0/200
	K14*#, K120, K249, K361	c.740_741CG>GA c.740C>G	p.P247R	0/200
*SPARC*	K15#	c.187G>A	p.E63K	0/200
	K34	c.276G>A	p.M92I	0/200
	K43	c.657C>A	p.D219E	0/200
	K66	c.747C>T	p.H249H	0/200
	K166	c.732C>T	p.D244D	0/200
	K188	c.204G>A	p.A68A	0/200
*SOD1*	K151	c.169+50delTAAACAG	-	0/200
	K283	c.169+50delTAAACAG	-	0/200

*Patients previous described [16]; ^#^ familial case; ^§^ novel mutation.

**Figure 2:**
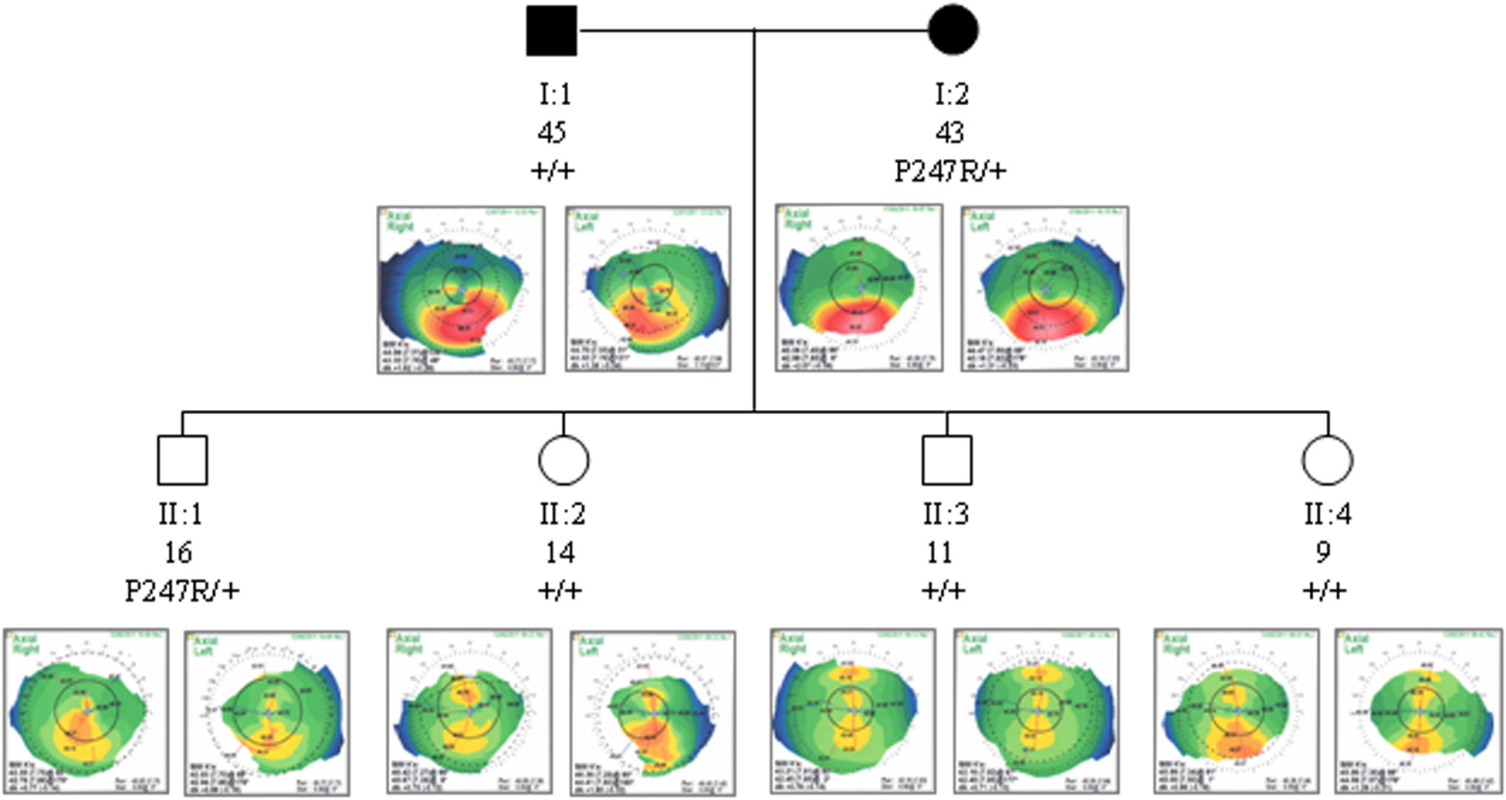
Segregation analysis of the *VSX1* mutation p.P247R in family K361. Each individual was reported by age (in years), genotype and videokeratographs. Filled symbols refer to keratoconus individuals, whereas open symbols are individuals without clinical keratoconus.

The p.P247R mutation in subjects K120 and K249 was due to the nucleotide change c.740C>G whereas in patients K14 and K361 was due to the substitution c.740_741CG>GA as previously reported by Heon et al. [15]. Segregation analysis of the p.P247R in the pedigree of patient K361 showed the presence of the variation in its first son, but the variation was absent in the remaining three child (Figure 2). Clinical phenotype of all family members was reported in Table 2. Two undescribed silent variants, c.348C>A (P116P) and c.474C>G (T158T) were identified in subjects K189 and K94, respectively.

### SPARC

Mutational screening of the nine coding exons (2-10) of *SPARC* was performed in all 302 patients. Six novel variants were detected, at heterozygous state, in six affected subjects. Three of them were silent variations c.204G>A (p.A68A), c.732C>T (p.D244D), and c.747C>T (p.H249H), while the remaining were non-synonymous nucleotide changes c.187G>A (p.E63K), c.276G>A (p.M92I), c.657C>A (p.D219E; Table 4). PolyPhen and SIFT tool analysis predicted p.E63K and p.D219E as benign and tolerated variants and were discordant for p.M92I (Table 2). The Position Specific Independent Counts (PSIC) score (2.023) indicated the methionine/isoleucine substitution as potentially deleterious, while the SIFT score (0.45) predicted this change as tolerated.

**Figure 3:**
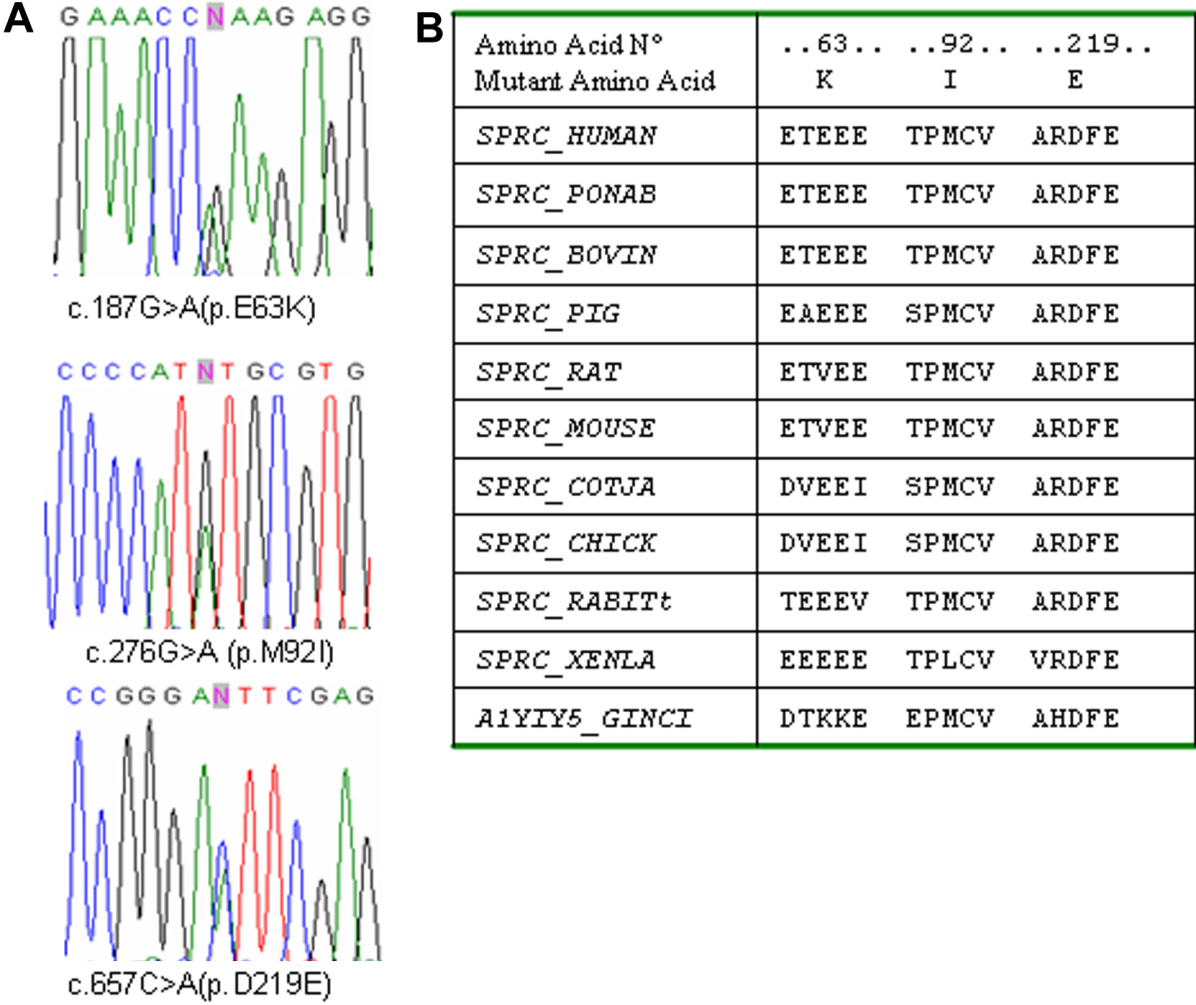
**A**: DNA sequence chromatogram of the *SPARC* variations: c.187G>A (p.E63K), c.276G>A (p.M92I), and c.657C>A (p.D219E; NM_003118). **B**: multiple sequence alignment of the amino acid sequences of SPARC in different species. Alignments were performed using the program Clustal.

The analysis of conservation across species showed that the aspartic acid at position 219 is the more well conserved amino acid residue; in X*enopus laevis* the methionine at position 92 is replaced by a leucine that has biochemical proprieties very similar to the isoleucine; while the glutamic acid at position 63 is the least conserved amino acid residue (Figure 3). None of the six variants were found, by DHPLC analysis, in the 200 control individuals. The p.E63K variant was inherited by patient K15 from his healthy mother, but the variant was absent in his keratoconic father who underwent penetrating keratoplasty. Additionally, the c.747C>T variant did not segregate with keratoconus in the pedigree of K66 patient (data not shown). No relatives of the other patients were available to study the possible segregation of the identified variants with the keratoconus. In addition, seven known single nucleotide polymorphisms (SNPs) rs7714314, rs2304052, rs2116780, rs2304051, rs1978707, rs41290587, and rs1053411 have been detected. The frequencies observed were compatible with the HapMap [39] information for Europeans.

### SOD1

**Figure 4:**
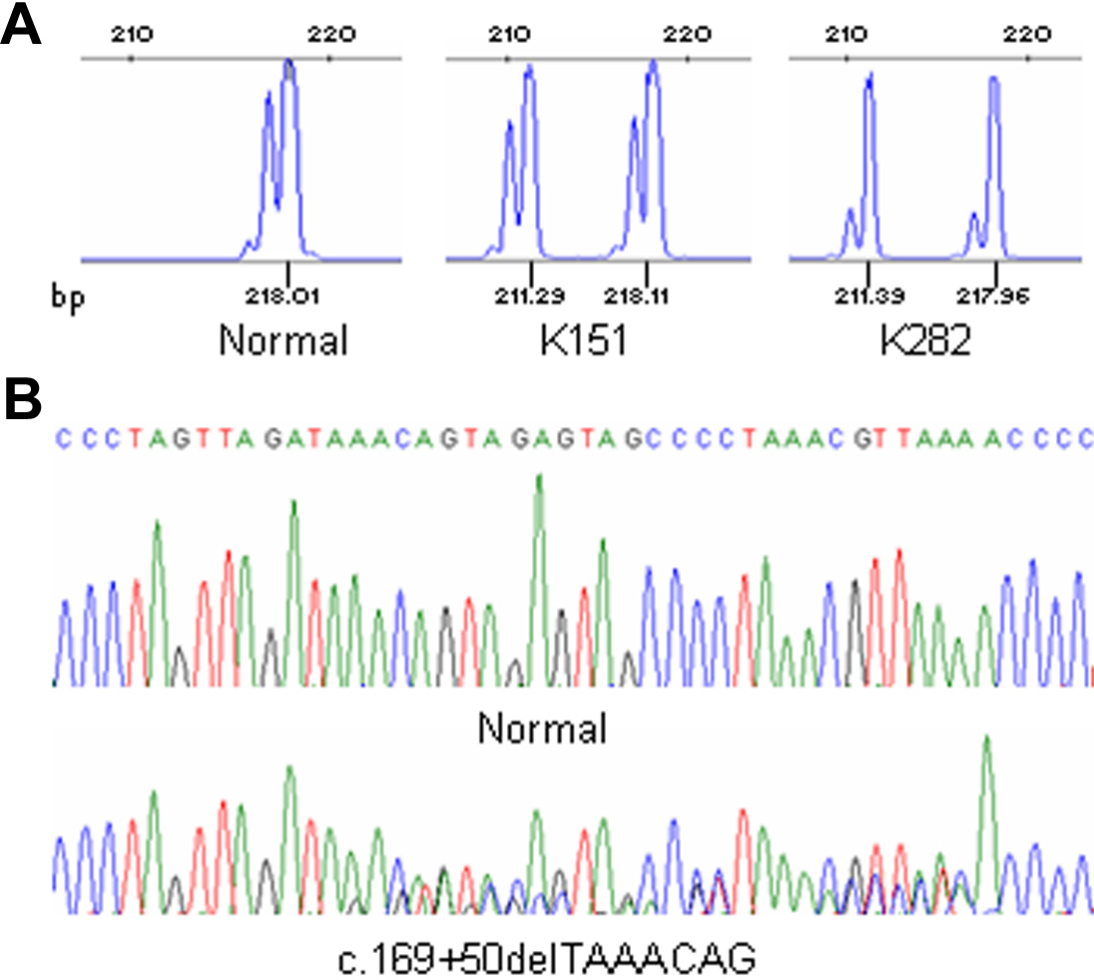
*SOD1* variation c.169+50delTAAACAG: fragments and sequencing analysis, in the patients K151 and K283, and normal control. **A**: DHPLC chromatograms showing the presence of the deleted allele of 211 bp in patients. **B**: DNA sequence electropherogram of the c.169+50delTAAACAG sequence variant in *SOD1* intron 2 (NM_000454).

Exon 2 and the flanking intronic regions of *SOD1* were PCR amplified using a fluorescent labeled primer. The fragments analysis allowed the identification of the 7-base deletion c.169+50delTAAACAG in two patients, K151 and K283, both affected by apparently sporadic keratoconus. This intronic deletion was absent in 200 controls. Figure 4 reports the electropherograms of the fragments and the sequencing analysis.

### TIMP3

No *TIMP3* mutations or novel variants were identified by sequencing of the 5 coding exons and the 516-bp fragment including part of the 5′ UTR, as well as CpG islands, putative binding sites for SP1, and a possible TATA box [34]. Two known SNPs were detected, rs9862 and rs11547635, showing alleles frequency compatible with HapMap data. Quantitative Methylation-Specific PCR was performed to determine methylation status of the *TIMP3* promoter region in three normal and three keratoconic corneal tissues. Methylation was not detected in any of the samples analyzed (data not shown).

### LOX

Sequencing of the whole coding sequence of *LOX* did not reveal novel variants. Two annotated SNPs were detected. The A allele of SNP rs41407546 (c.476C>A, p.P159Q) was found in heterozygosis in five individuals. Eight subjects were found homozygous and 78 were heterozygous for the A allele of SNP rs1800449 (c.473G>A, R158Q); the frequency of G allele (0.844) and A allele (0.155) were compatible with the HapMap [38] information for Europeans.

## Discussion

Keratoconus is a heterogeneous condition with variable clinical expression. Several hypotheses have been proposed concerning the genetic, environmental, biomechanical, and biochemical causes. In spite of a huge amount of research conducted to elucidate the etiology and the disease progression, *VSX1* is the sole gene indicated as an important genetic factor in determining the keratoconus. However, although the pathogenic role of *VSX1* is now accepted by many authors, only a small number of patients show mutations in this gene. Moreover, several loci for the disease have been mapped [9,28,39-47] and a large number of genes have been shown up- or down-regulated in keratoconic corneal tissues [30,48-58]. These observations confirm the genetic heterogeneity of the disease and also support the hypothesis that in some pedigrees the defect could be inherited as a multifactorial trait.

In this study, we evaluated a possible role played by five genes in determining keratoconus in a large cohort of individuals affected by sporadic or familial disease. The novel *VSX1*change c.715G>C (p.G239R) was found in one affected patient and in several unaffected relatives all showing corneal abnormalities at the videokeratography measurements.

The presence of this variant in unaffected relatives might suggest that it is a rare polymorphism without any pathogenic role. However the absence in 200 controls along with the PolyPhen and SIFT prediction and the high conservation across the species suggests it could be a pathogenic change. On the other hand we can not exclude that this variant could be a causative mutation with incomplete penetrance or low expressivity yielding corneal abnormalities in the unaffected relatives but causing significant damage in association with other genes or the environment in the affected patient.In addition, other previously reported amino acid substitutions p.L17P, p.D144E, p.P247R, p.G160D were identified in nine subjects (one familial and eight sporadic cases). These results demonstrate the incidence of *VSX1* mutant alleles in 5.9% (18 out of 302) among the cohort of Italian patients.

A large debate exists in the research community whether the *VSX1* variations identified in keratoconus have to be regarded as disease-related mutations or simply nonpathogenic variants.

Looking at the results obtained in previous studies [15-26], as well as in the present study, it is possible to summarize the main findings as follows:

- Six of the *VSX1* potential mutations, p.L17P, p.N151S, p.G160V, p.R166W, p.Q175H, and p.G239R were identified exclusively in keratoconus patients and in their relatives showing corneal abnormalities, but these mutations were absent in the controls.
- The p.G160D and p.P247R amino acid changes were found in keratoconus patients but they were also associated with mildly abnormal functions of the inner retina on electroretinography (ERG) examination and with Posterior Polymorphous Corneal Dystrophy [15]. In addition, since the p.G160D variation was absent in two out of four relatives with suspected keratoconus in an Italian family studied [16], its pathogenic role was not definitively clarified. None of the two variants has been identified in the controls.
- The p.H244R, p.L159M, and p.D144E variants were also found in three unaffected relatives and in seven out of 1312 (0.53%) controls analyzed. In addition, p.D144E did not show inheritance in two families [18,23]. For these reasons, all three *VSX1* variants may be considered as polymorphisms without pathogenic significance.
- Totally, *VSX1* sequence variations were found in 42 out of 1,350 (3.1%) keratoconus probands and in seven out of 1,412 (0.49%) controls.

In summary, even if there is controversial evidence for some variations, (i.e., p.D144E, p.L159M, p.G160D, and p.H244R), the role of *VSX1* in the pathogenesis of keratoconus cannot be absolutely excluded, as stated by some authors [19]. In fact, those variations also identified in healthy relatives of the patients and in the general population cannot be automatically considered simply as neutral polymorphism for several reasons. First, the “normal controls,” or unaffected relatives, carrying mutations apparently could be asymptomatic and may present subtle corneal abnormalities at the very end of the keratoconus phenotypical spectrum that could be revealed by pachymetric and posterior corneal surface maps, in addition to the videokeratography analysis [20]. Second, some variations among controls may represent incompletely penetrant mutations that could be pathogenic in another patient. Third, the segregation analysis performed by several authors supports either the autosomal dominant model of inheritance or the recessive one. Nevertheless, it cannot be excluded as the hypothesis of a major gene model and a multifactorial disease. In these latter cases, the *VSX1* changes found in keratoconus patients and in their normal relatives could represent genetic predisposition factors (a defect in one of the multiple factors needed to manifest the pathological phenotype).

SPARC is found widely in extracellular matrices and is predominantly expressed during embryogenesis and in adult tissues undergoing remodeling or repair. It is believed to play a modulator role in cell-cell and cell-matrix interactions, differentiation, ECM production and organization, wound healing, and angiogenesis [59-61]. In the eye, an increased SPARC level has been correlated with cataract [62], corneal wound repair [63], proliferative diabetic retinopathy [64], and glaucoma [65]. A possible role of *SPARC* in keratoconus has been hypothesized because of its function [27] and position [9]. Sequencing analysis of the gene allowed us to identify three novel variants leading to the amino acids substitutions p.E63K, p.M92I, and p.D219E. Data obtained by in silico analysis prediction and segregation analysis in the available pedigrees, though they are not sufficient to definitively rule out the involvement of *SPARC* in keratoconus, seem to suggest that the variations found have to be considered rare polymorphisms rather than causative mutations and further investigations are required.

SOD1 is a major cytoplasmic antioxidant enzyme that metabolizes superoxide radicals to molecular oxygen and hydrogen peroxide, thus providing a defense against oxygen toxicity [66]. *SOD1* mutations are associated with amyotrophic lateral sclerosis (ALS) [67,68]. A possible role in keratoconus was postulated because of its function and its chromosomal position. The presence of the c.169+50delTAAACAG change, the unique *SOD1* variant that was found associated to the keratoconus, was evaluated in the patients in this study. Udar et al. [32] identified this intronic deletion in two patients and demonstrated its segregation with keratoconus in only one of the two pedigrees studied. A subsequent study performed on 113 patients failed to identify the deletion [24]. In this study, the c.169+50delTAAACAG deletion was identified in two out of 302 patients. Both subjects appeared to be sporadic cases, and no relatives were included in the study. Taking into account the total number of unrelated patients studied, this *SOD1* variation was found in four out of 430 (0.9%), whereas it was absent in a total of 456 controls analyzed. Thus, a possible causative role of *SOD1* in the pathogenesis of keratoconus needs to be further studied.

*LOX* and *TIMP3* play a pivotal role in extracellular matrix maturation and remodeling, respectively, two process that could influence the loss of corneal structural components, leading to corneal thinning typical of keratoconus. Defects in *TIMP3* are the cause of Sorsby fundus dystrophy (OMIM 136900), a rare autosomal dominant macular disorder with age of onset in the fourth decade. Moreover, overexpression of *TIMP3* induces apoptosis in corneal stromal cell cultures [29]. Molecular analysis of both genes revealed no causative mutations in the cohort of patients but only known SNPs. Moreover, QMSP analysis performed in three keratoconic corneal tissues excluded methylation in the *TIMP3* promoter region. The results seem to exclude a possible role of these two genes in keratoconus.

In conclusion, in the present study, a mutational screening in a large cohort of unrelated keratoconus patients was performed and the results were analyzed. The results confirm the possible pathogenic role of *VSX1* though in a small number of patients, allow to exclude a possible involvement of *LOX* and *TIMP3* (at least in the study’s population), while not definitively clarifying the possible role played by *SOD1* and *SPARC* in determining the disease.

Further studies are required to identify other important genetic factors involved in the pathogenesis and progression of the disease which, in the authors’ opinion (and according to several other authors), should be considered a complex disease. As for many other complex traits, it is the time to shift the research approach to large consortiums combined with genome-wide association studies, with whole exome or entire genome sequencing. Such efforts will hopefully shed new light on the molecular genetics of the keratoconus.
